# Supplementary material for: Content-rich biological network constructed by mining PubMed abstracts
Source: BMC Bioinformatics. 2004 Oct 8;5:147. doi: 10.1186/1471-2105-5-147 (PMC528731; doi:10.1186/1471-2105-5-147)
Supplement: Additional File 5 — The original Chilibot query results of the term "long-term potentiation (LTP)" and 22 other terms, limiting the latest references analyzed to the years 1990, 1995, 2000, and 2004. [file 1471-2105-5-147-S5.bz2 › chilibotAdditionalFile5/ltp2004/html/ERK_CAMKII.html]

 


 **ERK** and **CAMKII** 
  
Found 20 abstracts in PubMed,  **20 abstracts were retrieved and analyzed**.  


---

 Search Google  |
 PDF files only 
|  EDU domain only 

---

**Interactive relationship** (e.g. stimulation, inhibition, etc)

- In two different cell lines, inhibition of  **CaMKII**  activity by specific inhibitors inhibited  **Erk**  1 2 phosphorylation.  Ref: 12954639 J Biol Chem, 2003
- Calcium calmodulin dependent protein kinase II  [ **CAMKII** ]  binds to Raf 1 and modulates integrin stimulated  **ERK**  activation.  Ref: 12954639 J Biol Chem, 2003
- the effects of  **ERK**  inhibition on migration may be mediated through its ability to inhibit  **CamKII**  activity.  Ref: 9925373 J Mol Cell Cardiol, 1998
- Additionally, we provide evidence that GLP1 stimulated activation of  **Erk**  requires an influx of calcium through L type voltage gated calcium channels and the activation of calcium calmodulin dependent protein kinase II  [ **CAMKII** ] .  Ref: 12364324 J Biol Chem, 2002
- Although KN62 did not affect ERK2 activity in response to PDGF, PD98059 markedly inhibited PDGF stimulated  **CamKII**  activity, suggesting that activation of  **CamKII**  by PDGF was dependent on  **ERK**  activity.  Ref: 9925373 J Mol Cell Cardiol, 1998
- Inhibition of neuronal differentiation by nuclear  **CaMKII**  was independent of MAPK, as sustained  **Erk**  phosphorylation was not affected.  Ref: 12570987 Am J Physiol Cell Physiol< MedlineTA>Am J Physiol Cell Physiol, 1998

**Parallel relationship** (e.g. studied together, co-existance, homology, etc.)

- Circadian modulation of ligand affinity is a posttranslational effect and is driven by rhythms in the activities of two protein kinases  **Erk**  and calcium calmodulin dependent protein kinase II  **CaMKII** .  Ref: 11182096 Neuron, 2001
- Group I metabotropic glutamate receptors control phosphorylation of CREB, Elk 1 and  **ERK**  via a  **CaMKII**  dependent pathway in rat striatum.  Ref: 11682144 Neurosci Lett, 2001
- In immunohistochemical study, KA dramatically increased the phosphorylation of extracellular signal regulated protein kinase p  **ERK** , c Jun N terminal kinase 1 p JNK1, and calcium calmodulin dependent protein kinase II  [ **CAMKII** ]  p CaMK II.  Ref: 12788213 Brain Res Bull< MedlineTA>Brain Res Bull, 2001
- Inhibition of either  **CaMKII**  or the MEK  **ERK**  pathway, both of which phosphorylate MAP2, inhibits dendrite formation, but inhibition of both pathways simultaneously is required for dendrites to retract.  Ref: 12086645 Neuron, 2002
- We conclude that lead activates PKC,  **CaMKII**  or MEK  **ERK**  p90RSK pathways in GT1 7 cells, leading to CREB phosphorylation and modulation of gene expression.  Ref: 12832008 Brain Res Bull, 2003
- This is the first demonstration that  **CaMKII**  interplays with Raf 1 and regulates  **Erk**  activation induced by Ras stimulated Raf 1.  Ref: 12954639 J Biol Chem, 2003
- To evaluate putative roles of calcium calmodulin dependent protein kinase II  **CaMKII**  in CREB, Elk 1 and  **ERK**  phosphorylation, the  **CaMKII**  inhibitor, KN62, was infused simultaneously with the group I mGluR agonist, dihydroxyphenylglycine DHPG, into the rat dorsal striatum.  Ref: 11682144 Neurosci Lett, 2001
- Four protein kinase cascades have garnered considerable attention in the study of information storage at both the synaptic and behavioral levels Ca phospholipid dependent protein kinase PKC, Ca calmodulin dependent protein kinase II  **CaMKII** , cAMP dependent protein kinase PKA, and extracellular signal regulated kinase  **ERK** .  Ref: 11954557 Neuroscientist, 2002
- In this study, we demonstrate that integrin stimulation by fibronectin FN, besides activating the Ras  **Erk**  pathway, generates an auxiliary calcium signal that activates calmodulin and the calcium calmodulin dependent protein kinase II  **CaMKII** .  Ref: 12954639 J Biol Chem, 2003
- These results suggest that  **ERK**  and  **CaMKII**  positively regulate the D2R promoter.  Ref: 12421350 J Neurochem, 2002
- These results indicate that lead activated PKC,  **CaMKII**  and MEK  **ERK**  p90RSK pathways simultaneously, all of which contributed to CREB phosphorylation.  Ref: 12832008 Brain Res Bull, 2003
- Phosphorylation consensus sites for various kinases exist within the sequence of the potassium channel subunit Kv4.2, including consensus sites for extracellular signal regulated kinase mitogen activated protein kinase  **ERK**  MAPK, protein kinase A PKA, protein kinase C PKC, and calcium calmodulin dependent kinase II  **CaMKII** , and kinase assays have shown that particular amino acids of the consensus sites are bonafide phosphorylation sites in vitro.  Ref: 11040264 Learn MemLearn Mem, 2003
- In calcium clamped skinned pulmonary arterial strips from newborn rabbits, the anesthetics induce relaxation, which is prevented by the PKC inhibitors MEK  **ERK**  12,  **CaMKII** , and p38.  Ref: 12826852 Anesthesiology, 2003
- Additionally, the activations of PKC and p38... calcium calmodulin dependent protein kinase II  [ **CAMKII** ] , and  **ERK** , by C2 ceramide play important regulatory roles in C2 ceramide induced proENK mRNA expression via activating the CREB.  Ref: 11382404 Brain Res, 2001
